# Benign Metastasizing Leiomyomatosis: The Paradox of a Benign Tumor That Spreads

**DOI:** 10.51894/001c.164060

**Published:** 2026-08-01

**Authors:** Sushmita Mantravadi, Kesava Achuta Manikanta, Sai Tharun Reddy Gopannagari, Furqan Siddiqi, Pranay Gupta

**Affiliations:** 1 Garden City Hospital

## 30

### Introduction

Benign Metastasizing Leiomyomatosis (BML) is a rare condition characterized by dissemination of histologically benign uterine leiomyoma to distant sites, most commonly the lungs, but also to other organs such as lymph nodes, bones, soft tissues, and the skin, without malignant features on pathology. To date, fewer than 200 cases have been reported worldwide.

### Case Presentation

We present a case of 40-year old female with a past medical history of uterine fibroid, mucinous cystadenoma and mature cystic teratoma of left ovary status post left salpingo oophorectomy who presented with shortness of breath. On arrival,she was febrile to 103.2°F with pulse of 121, respiratory rate of 20 and saturating at 94% on room air. Physical exam was unremarkable except for mild hypoxemia.Chest imaging revealed multiple bilateral varied sized pulmonary nodules,the largest being 2.5 cm without a new focal infiltrate. She required supplemental oxygen and was started empirically on broad spectrum antibiotics which were later discontinued after negative blood cultures. A video-assisted thoracoscopic biopsy demonstrated multiple nodules composed of smooth muscle proliferation positive for desmin, estrogen and progesterone receptors indicative of BML and no evidence of malignancy on histopathology. She was referred for outpatient gynecology and endocrinology follow up to discuss hormonal suppression therapy for stabilizing or reducing tumor burden.

### Discussion

BML typically affects women with a history of uterine leiomyomas, often following surgical procedures like myomectomy or hysterectomy. The metastatic lesions are composed of smooth muscle cells that morphologically benign, showing low mitotic activity, minimal or no atypia, and strong positivity for estrogen and progesterone receptors, mirroring the primary uterine tumor. Even though the pathogenesis is not fully understood, the proposed mechanisms include hematogenous or lymphatic, or intravascular extension or mechanical displacement during surgery resulting in surgically induced vascular dissemination. Despite their metastatic behavior, they lack the cytologic features of malignancy seen in leiomyosarcoma and generally have an indolent course. Gonadotropin-releasing hormone analogs and aromatase inhibitors have shown promise in managing PBML. Bilateral oophorectomy can lead to regression of pulmonary lesions. This case underscores the importance of considering BML in women with pulmonary nodules and a history of uterine fibroids.

